# Sex-dependent dynamic changes in pectoral muscle properties and gut microbiota composition in Qiandongnan Xiaoxiang chickens

**DOI:** 10.3389/fmicb.2026.1873217

**Published:** 2026-07-10

**Authors:** Meilin Hao, Wenjie Cheng, Junhong Zhu, Lanlan Yi, Zhaohong Zhou, Qiangfei Li, Sumei Zhao, Yuxiao Xie

**Affiliations:** 1 College of Biology and Agriculture, Zunyi Normal College, Zunyi, Guizhou, China; 2 Faculty of Animal Science and Technology, Yunnan Agricultural University, Kunming, China; 3 First People's Hospital of Kunming City, Kunming, China; 4 Zunyi Animal Husbandry and Fishery Station, Zunyi, Guizhou, China; 5 Xishui Bureau of Agriculture and Rural Affairs, Zunyi, Guizhou, China

**Keywords:** amino acid, gut microbiota, muscle fiber, Qiandongnan Xiaoxiang chicken, sex difference

## Abstract

**Introduction:**

Sex differences profoundly affect meat quality traits in chickens, but the role of gut microbiota in this process remains unclear, especially in indigenous breeds.

**Methodology:**

This study investigated pectoral muscle fiber characteristics, amino acid composition, and cecal microbiota of Qiandongnan Xiaoxiang chickens at 60,120, and 180 days of age (*n* = 6 per sex per age).

**Results:**

At 60 days, roosters had higher total fiber number and density, while hens showed larger fiber area, average area, and diameter (*P* < 0. 05). At 120 days, hens maintained larger total fiber area (*P* < 0. 05); at 180 days, total fiber area remained higher in hens (*P* < 0. 05). Amino acid content shifted from hen dominance at 60/120 days to rooster dominance at 180 days. Gut microbiota diversity and composition showed significant sex differences, with differential genera decreasing from 26 (60d) to2 (180d). Correlation analysis linked *Phascolarctobacterium* and *Intestinimonas* positively with muscle traits.

**Discusssion:**

These findings reveal dynamic sexual dimorphism in muscle properties and gut microbiota, suggesting a potential association between sex-dependent microbiota variation and muscle-related traits.

## 1 Introduction

Chicken is one of the most consumed meats globally and serves as an important source of animal protein (Cai et al., 2024). Due to its characteristics of high protein, low fat, and low cholesterol, along with its affordable price, the poultry industry has become the fastest-growing livestock subsector (Shioda et al., 2024). Currently, research on the sensory characteristics, nutritional value, and flavor traits of chicken is increasing. This has also promoted the trend of analyzing the mechanisms of meat-quality-related trait formation at the molecular level (Alsoufi et al., 2022). Qiandongnan Xiaoxiang chicken is a local specialty breed from Guizhou Province in China. It is renowned for its excellent meat quality and flavor, good environmental adaptability, and strong stress resistance, and has been included in the Chinese Livestock and Poultry Genetic Resources Catalog (Hao et al., 2025; Tian et al., 2025). Deeply elucidating the biological basis affecting the meat-quality-related traits of Qiandongnan Xiaoxiang chicken is of great significance for the conservation and utilization of this breed resource.

Muscle growth and development is a complex biological process regulated by multiple factors including genetics, nutrition, environment, and endogenous hormones (Magagula et al., 2024; Volkova et al., 2024; Xu et al., 2025). Among the numerous factors affecting meat quality traits, sex difference is an important factor. Numerous studies have shown significant differences between male and female chickens in growth rate, feed conversion rate, slaughter performance, and meat quality. Compared with hens, roosters exhibit higher final live weight and lower feed conversion rates (Trocino et al., 2015). These differences in growth performance and meat quality indicators caused by sex further confirm that sex is a key biological factor regulating muscle development and meat-quality-related phenotypes. Muscle fiber morphology affects tenderness and juiciness (Listrat et al., 2016), while amino acid composition (especially essential and flavor-related amino acids) determines nutritional value and taste (Yin et al., 2023). Moreover, these two traits are closely interrelated during muscle growth and development (Sato et al., 2018). Therefore, simultaneous analysis of muscle fiber morphology and amino acid content provides a solid basis for understanding the biological foundation of meat quality formation.

As a complex micro-ecosystem within the host, the gut microbiota not only participates in the digestion and absorption of nutrients but also interacts with the host through its metabolites, regulating energy metabolism and protein synthesis in distal tissues (de Vos et al., 2022; Martin-Del-Campo et al., 2023). The proposal of the “gut-muscle axis” concept reveals a close functional connection between intestinal microorganisms and muscle metabolism. Studies have confirmed that changes in gut microbiota structure can affect the efficiency of muscle protein synthesis and the transformation of muscle fiber types, thereby influencing the meat-quality-related traits (Gao et al., 2025; Yan et al., 2025). There is significant sexual dimorphism in the composition and function of the gut microbiota (Ghaffar et al., 2025). Sex hormones can indirectly shape the host's microbial community structure by influencing intestinal mucosal immunity, altering intestinal permeability, and affecting the profile of microbial metabolites (Yoon and Kim, 2021; Tang et al., 2025). This suggests that the function of the “gut-muscle axis” may be regulated by sex factors. Clinical studies have also found that the association between gut microbiota and sarcopenia exhibits different microbial characteristics in different sexes (Aliwa et al., 2023). Direct evidence from poultry studies also supports sex-dependent differences in cecal microbiota composition, with male chickens exhibiting higher abundance of *Bacteroides, Megamonas*, and *Lactobacillus*—genera involved in glycan metabolism and muscle development—while female chickens show enrichment of *Enterococcus* and *Ruminococcaceae*, which are associated with lipid metabolism (Cui et al., 2021). These findings provide important clues for analyzing the mechanisms of sex differences in meat-quality-related traits from the perspective of the “gut-muscle axis.”

The interaction between microbial community structure and sex factors on meat-quality-related traits of Qiandongnan Xiaoxiang chicken has not been systematically reported. In this study, intestinal contents and pectoral samples were collected from male and female Qiandongnan Xiaoxiang chickens at 60, 120, and 180 days of age. 16S rRNA sequencing technology, muscle fiber histological analysis, and amino acid content analysis we systematically compared the differential characteristics of gut microbiota structure, muscle fiber properties, and amino acid content between sexes at different ages. This study focusing on analyzing the correlation patterns between microbial changes and muscle metabolic phenotypes. The specific hypotheses of this study are: (1) sex affects muscle fiber morphology and amino acid profiles in an age-dependent manner, as reflected by a significant sex × age interaction; and (2) gut microbiota composition is associated with these sex-dependent muscle phenotypes, potentially via the gut-muscle axis. The results will provide insights into sex-dependent regulation of gut-muscle axis function and offer a correlational basis for understanding the biological foundation of meat-quality-related traits in local chicken breeds. However, causal inferences cannot be drawn from this observational study, and further mechanistic validation is needed.

## 2 Materials and methods

### 2.1 Animals and sample collection

A total of 201-day-old Qiandongnan Xiaoxiang chickens (obtained from the Qiandongnan Xiaoxiang Chicken Breeding Farm, Guizhou Province, China) were used in this experiment. The chickens were raised in the same environmentally controlled house with a floor pen system. The pens were made of galvanized wire, each measuring 2.0 m (length) × 1.5 m (width) × 0.8 m (height). Males and females were reared in separate pens (10 pens per sex, 10 birds per pen). The environmental conditions were maintained as follows: temperature was kept at 33 °C for the first week and then reduced by 2–3 °C per week until reaching 22 °C; relative humidity was maintained at 55–65%; and a lighting schedule of 23 h light and 1 h dark was applied during the first week, followed by 16 h light and 8 h dark thereafter. All birds were fed the same basal diets under the same feeding program, consisting of a starter phase (1–56 days) and a grower phase (57–180 days). The composition and nutritional levels of the basal diets are shown in Table 1. The diets differed between the two phases (starter vs. grower), which is standard practice for age-appropriate nutrition. At 60, 120, and 180 days of age, six male and six female chickens were randomly selected from the pooled chickens of the corresponding sex. This sample size is consistent with previous poultry gut microbiota studies (Du et al., 2024). We selected individuals whose body weight was within ± 10% of the group mean. One bird was selected from each pen, and the same pen was not sampled again at different time points to avoid repeated sampling. Before slaughter, birds were fasted for 12 h with free access to water. Slaughtering, cecal content collection, and pectoral muscle sampling were performed synchronously for all birds at each age. Pectoral tissue and cecal contents were collected and immediately snap-frozen in liquid nitrogen. Additionally, portions of pectoral tissue samples were snap-frozen in liquid nitrogen and stored at −80°C for subsequent amino acid content analysis. All animal procedures in this study were approved by the Animal Protection and Utilization Institutional Committee of Zunyi Normal College (Approval No. ZYNU-FA-2024-06).

**Table 1:** The ingredients and nutritional level of basic diet.

Items	Stage (1–56d)	Stage (57–180d)
Ingredients (%)		
Corn	59.32	66.50
Soybean meal	26.50	18.50
Corn gluten meal	3.03	3.20
Wheat bran	4.73	4.00
Soybean oil	2.36	3.93
L-lysine.HCl	0.30	0.32
DL-methionine	0.12	0.10
CaHPO_4_	1.41	1.30
Limestone	1.08	0.85
NaCl	0.15	0.30
Premix^1^	1.00	1.00
Total	100	100
Nutrition level^2^		
Crude protein	18.97	16.03
Metabolizable energy(MJ/kg)	12.34	12.95
Lysine	1.05	0.90
Methionine	0.73	0.63
Calcium	0.91	0.78
Available phosphorus	0.38	0.35

Supplied per kilogram of diets: vitamin A, 6, 000 IU; thiamine, 2.00 mg; riboflavin, 4.00 mg; niacin, 42.00 mg; pyridoxine, 4.00 mg; cobalamin, 0.01 mg; vitamin D_3_, 2, 000 IU; vitamin E, 30 IU; vitamin K_3_, 1.80 mg; calcium pantothenate, 10.00 mg; biotin, 0.15 mg; folic acid, 0.85 mg; Fe, 80.00 mg; Cu, 8.00 mg; Mn, 80.00 mg; Zn, 65.00 mg; I, 0.50 mg; Se, 0.25 mg.^2^Crude protein was a determined value, while the rest were calculated values.

### 2.2 HE staining

Pectoral muscle samples were collected from the same anatomical location (the central region of the left pectoralis major) of each chicken, fixed in 4% formaldehyde solution for 24 h, embedded in paraffin, and sliced into 5-μm thick sections; three non-serial sections per chicken were prepared and stained with hematoxylin and eosin (H&E). For each section, five randomly selected, non-overlapping fields were captured at 400 × magnification using a light microscope (Olympus BX53, Tokyo, Japan), and images were analyzed with Image-Pro Plus 6.0 software (Media Cybernetics, Rockville, MD, USA). The following parameters were quantified: muscle fiber diameter (mean diameter of individual fibers measured across the minor axis), average muscle fiber area (mean cross-sectional area of individual fibers), total muscle fiber area (sum of cross-sectional areas of all fibers within a field), total number of muscle fibers (count of fibers per field), and muscle fiber density (number of fibers per unit area, calculated as total number of muscle fibers divided by the field area in mm^2^). For each chicken, the values obtained from all fields (3 sections × 5 fields = 15 fields) were averaged to yield a single representative value per parameter, and all analyses were performed in a blinded manner regarding sex and age groups.

### 2.3 Amino acid content determination

Amino acids were determined using an automatic amino acid analyzer (membraPure, Germany, A300) with post-column derivatization. Approximately 100 mg of sample was weighed into a 20 mL hydrolysis tube, and 10 mL of 6 mol/L hydrochloric acid hydrolysis solution was added, followed by the addition of phenol. The hydrolysis tube was placed in a freezing agent and frozen for 5 min. After repeated evacuation and nitrogen flushing three times, the tube was hydrolyzed in a constant-temperature drying oven at 110°C for 24 h. After cooling, mixing, and filtration, an appropriate volume of the filtrate was transferred into a concentrator and evaporated under vacuum at 60°C. Sodium citrate buffer solution was added, mixed well, and centrifuged at 4,000 r/min. The supernatant was collected for analysis (Davidson, 1997).

### 2.4 16S rRNA sequencing

Microbial DNA was extracted using HiPure Stool DNA Kits (Magen Biotech, China). DNA concentration was determined using a NanoDrop 2,000 (Thermo Fisher Scientific, USA), and DNA integrity was assessed by agarose gel electrophoresis. The V3–V4 hypervariable region of the 16S rRNA gene was amplified using primers 341F (CCTACGGNGNGCWGCAG) and 806R (GGACTACHVGGGTATCTAAT) (Guo et al., 2017). Sequencing libraries were constructed using the Illumina DNA Prep Kit (Illumina, USA). Library quality was assessed using the ABI StepOnePlus Real-Time PCR System (Thermo Fisher Scientific, USA). Qualified libraries were pooled and sequenced on the NovaSeq 6,000 platform (Illumina, USA) using the PE250 mode.

### 2.5 Sequencing data analysis

Raw data were filtered using FASTP (v 0.18.0) to obtain clean reads (Chen et al., 2018). The filtering criteria were as follows: removal of reads containing ≥10% unknown nucleotides (N), removal of reads with ≥50% of bases having a Phred quality score ≤ 20, and removal of reads containing adapters. Clean reads were then merged into tags using FLASH (v 1.2.11) (Magoc and Salzberg, 2011) with a minimum overlap of 10 bp and a maximum mismatch rate of 2%. Low-quality tags were filtered to obtain clean tags (Bokulich et al., 2013). Tags were truncated at the first base where the continuous low-quality (quality threshold ≤ 3) reached a length of 3 bp, and only tags with contiguous high-quality bases longer than 75% of the tag length were retained. Operational taxonomic units (OTUs) were clustered from clean tags at 97% similarity threshold using Usearch (v 11.0.667) (Edgar, 2010). Chimeric sequences were removed using UCHIME, and the resulting effective tags were used for OTU abundance statistics (Edgar et al., 2011). Representative OTU sequences were aligned against the SILVA database (v 138.2) (Pruesse et al., 2007), and taxonomic classification was performed using the RDP classifier software (v 2.14) (Wang et al., 2007). Alpha diversity (Chao1 and ACE) was analyzed by two-way ANOVA (sex × age) after Shapiro–Wilk normality test, followed by Tukey's HSD or Kruskal–Wallis with Dunn's test and FDR correction for non-normal data. Beta diversity was assessed by PCoA (Bray-Curtis and Jaccard) and PERMANOVA (adonis2, sex × age), with pairwise PERMANOVA and FDR correction. Differential genera were identified by Welch's t-test with FDR correction. All analyses were performed in R (vegan and RVAideMemoire packages).

### 2.6 Correlation analysis between microbiota and muscle phenotypes

The top 50 most abundant genera based on mean relative abundance across all samples were selected and subjected to Spearman correlation analysis with muscle traits (muscle fiber characteristics and amino acid content).

### 2.7 Statistical analysis

Results are expressed as mean ± standard deviation (SD) unless otherwise stated. Statistical analysis was performed using SPSS 20.0 software. Muscle fiber characteristics and amino acid contents were analyzed using two-way ANOVA with sex and age as fixed factors. The *P*-values for sex, age, and their interaction were calculated. Where significant interactions were detected, simple effect analyses with Bonferroni correction were performed to compare sexes within each age. *P* < 0.05 was considered statistically significant.

## 3 Results

### 3.1 Effects of sex on pectoral fiber characteristics in Qiandongnan Xiaoxiang chickens

**Figure 1:**
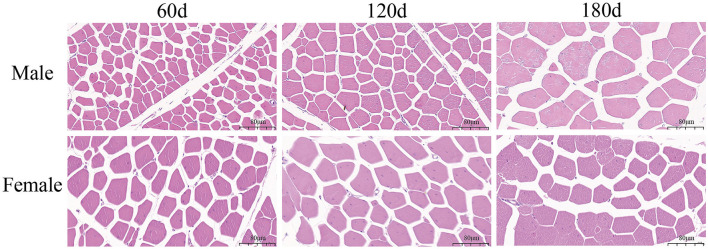
HE staining of pectoral tissue in Qiandongnan Xiaoxiang chickens (400 × ). Scale bar = 80 μm. *n* = 6 per sex per age group.

We compared the effects of sex on pectoral fiber characteristics in Qiandongnan Xiaoxiang chickens at different ages, and the results are shown in Table 2, along with the P-values for sex × age interaction from two-way ANOVA. Two-way ANOVA revealed no significant sex × age interaction for any of the muscle fiber traits (all *P* > 0.05), indicating that the sex effects were largely consistent across ages. At 60 days of age, the total number of muscle fibers and muscle fiber density were significantly higher in roosters than in hens (*P* < 0.05); whereas the total muscle fiber area, average muscle fiber area, and muscle fiber diameter were significantly greater in hens than in roosters (*P* < 0.05). At 120 days of age, total muscle fiber area remained significantly greater in hens than in roosters (*P* < 0.05), while no significant differences were observed between sexes in total number of muscle fibers, fiber density, or fiber diameter. At 180 days of age, only total muscle fiber area remained significantly greater in hens than in roosters (*P* < 0.05), with no significant differences in the other indicators between sexes (Figure 1). With increasing age, total number of muscle fibers and fiber density showed a decreasing trend in both roosters and hens, while total muscle fiber area, average muscle fiber area, and fiber diameter showed an increasing trend. Sex differences were mainly reflected in the following: at 60 days of age, all indicators showed significant differences, with hens having larger individual muscle fibers but fewer in number; at 120 and 180 days of age, the difference in total muscle fiber area persisted.

**Table 2:** Comparison of pectoral fiber parameters between male and female Qiandongnan Xiaoxiang chickens at different ages.

Index	60d		120d		180d		*P* (sex × age)
	Male	Female	Male	Female	Male	Female	
Total number of muscle fibers (n)	149 ± 30.23^a^	99.17 ± 28.05^b^	97.67 ± 26.40	74.5 ± 39.71	54.5 ± 15.78	61.17 ± 21.84	0.0554
Total muscle fiber area (mm^2^)	0.064 ± 0.011^a^	0.083 ± 0.0057^b^	0.070 ± 0.010^a^	0.085 ± 0.0048^b^	0.076 ± 0.0074^a^	0.089 ± 0.0048^b^	0.5156
Average muscle fiber area (mm^2^)	0.00044 ± 0.000029^a^	0.00089 ± 0.00023^b^	0.00074 ± 0.00014	0.0014 ± 0.00052	0.0015 ± 0.00032	0.0016 ± 0.00047	0.2650
Muscle fiber density (n/mm^2^)	1220.91 ± 247.78^a^	812.57 ± 229.81^b^	800.28 ± 216.34	610.46 ± 325.41	446.57 ± 129.33	501.20 ± 178.95	0.0554
Muscle fiber diameter (mm)	0.028 ± 0.0019^a^	0.040 ± 0.0057^b^	0.038 ± 0.0033	0.045 ± 0.011	0.056 ± 0.0080	0.053 ± 0.0081	0.0573

Data are presented as mean ± SD. *n* = 6 per sex per age group. Within rows with no common superscripts differ significantly (*P* < 0.05).

### 3.2 Comparison of amino acid contents in pectorals of Qiandongnan Xiaoxiang chickens

Two-way ANOVA revealed significant sex × age interactions for most amino acids (*P* < 0.05), except for alanine and tyrosine (*P* > 0.05), indicating that the sex difference in amino acid profiles changed dynamically with age. At 60 days of age, the contents of aspartic acid, glutamic acid, and phenylalanine in the pectorals of hens were significantly higher than those of roosters (*P* < 0.05). By 120 days of age, this amino acid advantage in hens further expanded, with significantly higher contents of arginine, aspartic acid, glutamic acid, glycine, isoleucine, leucine, lysine, phenylalanine, proline, threonine, and valine compared to roosters (*P* < 0.05), while histidine content was significantly lower than in roosters (*P* < 0.05). At 180 days of age, the amino acid enrichment pattern reversed, with roosters exhibiting significantly higher contents of glutamic acid, isoleucine, leucine, phenylalanine, and valine than hens (*P* < 0.05). At 60 days of age, the contents of branched-chain amino acids (BCAA), essential amino acids (EAA), non-essential amino acids (NEAA), and total amino acids (Total AA) in the pectoral muscle of hens were all higher than those of roosters, with EAA reaching significant levels (*P* < 0.05). At 120 days of age, hens had significantly higher contents of all four amino acid categories than roosters (*P* < 0.05). At 180 days of age, roosters had signicantly higher contents of BCAA, EAA, and Total AA than hens (*P* < 0.05) (Table 3). Overall, these results demonstrate a dynamic reversal of sex advantage in amino acid profiles from hens (at 60 and 120 days) to roosters (at 180 days).

**Table 3:** Amino acid composition of pectorals in Qiandongnan Xiaoxiang chicken.

Amino acids (mg/g)	60d		120d		180d		*P* (sex × age)
	Male	Female	Male	Female	Male	Female	
Aspartic acid	55.98 ± 4.57^a^	65.73 ± 5.24^b^	65.64 ± 5.35^a^	80.38 ± 7.75^b^	85.03 ± 1.92	81.27 ± 3.02	0.0005
Threonine	16.62 ± 1.19	20.04 ± 2.9	20.19 ± 2.38^a^	35.31 ± 0.91^b^	34.94 ± 1.56	33.33 ± 1.31	0.0001
Serine	23.83 ± 2.01	27.54 ± 2.42	28.06 ± 2.22	29.93 ± 0.89	30.47 ± 0.6	29.81 ± 0.97	0.0147
Glutamic acid	76.9 ± 6.09^a^	90.85 ± 8.97^b^	92.55 ± 7.99^a^	136.73 ± 2.92^b^	142.05 ± 3.46^a^	136.84 ± 1.68^b^	0.0001
Glycine	33.66 ± 6.27	32.24 ± 1.98	31.88 ± 3.14^a^	36.68 ± 1.57^b^	36.83 ± 3.33	32.35 ± 1.11	0.0044
Alanine	42.47 ± 3.99	46.34 ± 3.48	46.69 ± 4.75	44.94 ± 2.65	47.59 ± 5.99	47.37 ± 1.49	0.2395
Valine	1.09 ± 0.58	0.90 ± 0.85	0.39 ± 0.11^a^	41.36 ± 1.12^b^	43.18 ± 1.14^a^	40.73 ± 1.3^b^	0.0001
Isoleucine	9.6 ± 1.98	10.96 ± 1.77	10.85 ± 1.34^a^	39.96 ± 0.94^b^	41.44 ± 1.2^a^	39.07 ± 1.1^b^	0.0001
Leucine	29.07 ± 14.52	41.11 ± 4.32	41.83 ± 3.65^a^	64.19 ± 1.67^b^	66.73 ± 1.55^a^	63.71 ± 1.42^b^	0.0003
Tyrosine	24.99 ± 15.13	22.73 ± 1.96	23.07 ± 1.75	24.09 ± 0.95	24.59 ± 0.69	24.49 ± 1.14	0.8233
Phenylalanine	16.96 ± 2.43^a^	21.35 ± 2.25^b^	21.6 ± 2.05^a^	31.79 ± 0.88^b^	32.47 ± 0.76^a^	31.21 ± 0.68^b^	0.0001
Histidine	52.8 ± 5.3	57.54 ± 2.78	59.58 ± 2.4^a^	54.87 ± 2.84^b^	57.08 ± 1.62	54.48 ± 2.41	0.0009
Lysine	36.7 ± 3.01	42.99 ± 5.29	43.76 ± 4.18^a^	69.79 ± 3.12^b^	73.06 ± 3.59	68.74 ± 3.03	0.0001
Arginine	30.15 ± 2.23	34.29 ± 3.29	34.62 ± 2.71^a^	57.01 ± 3.89^b^	60.29 ± 2.05	57.73 ± 1.23	0.0001
Proline	20.86 ± 2.6	23.36 ± 4.7	26.59 ± 2.6^a^	34.1 ± 1.44^b^	34.3 ± 1.81	30.72 ± 2.74	0.0007
BCAA	39.76 ± 12.95	52.97 ± 6.07	53.08 ± 4.90^a^	145.51 ± 3.63^b^	151.35 ± 3.69^a^	143.51 ± 3.78^b^	0.0001
EAA	193.00 ± 15.57^a^	229.18 ± 21.63^b^	232.82 ± 17.36^a^	394.28 ± 9.75^b^	409.19 ± 11.28^a^	388.99 ± 8.39^b^	0.0001
NEAA	278.69 ± 33.42	308.78 ± 23.30	314.48 ± 22.25^a^	386.86 ± 11.83^b^	400.86 ± 15.75	382.84 ± 5.42	0.0002
Total AA	471.69 ± 39.83	537.96 ± 44.33	547.30 ± 38.96^a^	781.14 ± 21.20^b^	810.04 ± 26.50^a^	771.84 ± 12.94^b^	0.0001

Values are mean ± SD. *n* = 6 per sex per age group. Within rows with no common superscripts differ significantly (*P* < 0.05).

### 3.3 Sequencing depth and quality control

After quality filtering and chimera removal, the effective tags per sample ranged from 59,515 to 1,02,325, and the effective ratio (effective tags/raw reads) ranged from 78.61% to 85.10%. The N50 value was 461 bp for the vast majority of samples, indicating high assembly quality. The number of observed OTUs per sample ranged from 2,360 to 3,952. Detailed per-sample metrics are provided in Supplementary Table 1.

### 3.4 Comparison of gut microbial diversity in different sexes of Qiandongnan Xiaoxiang chickens

**Figure 2:**
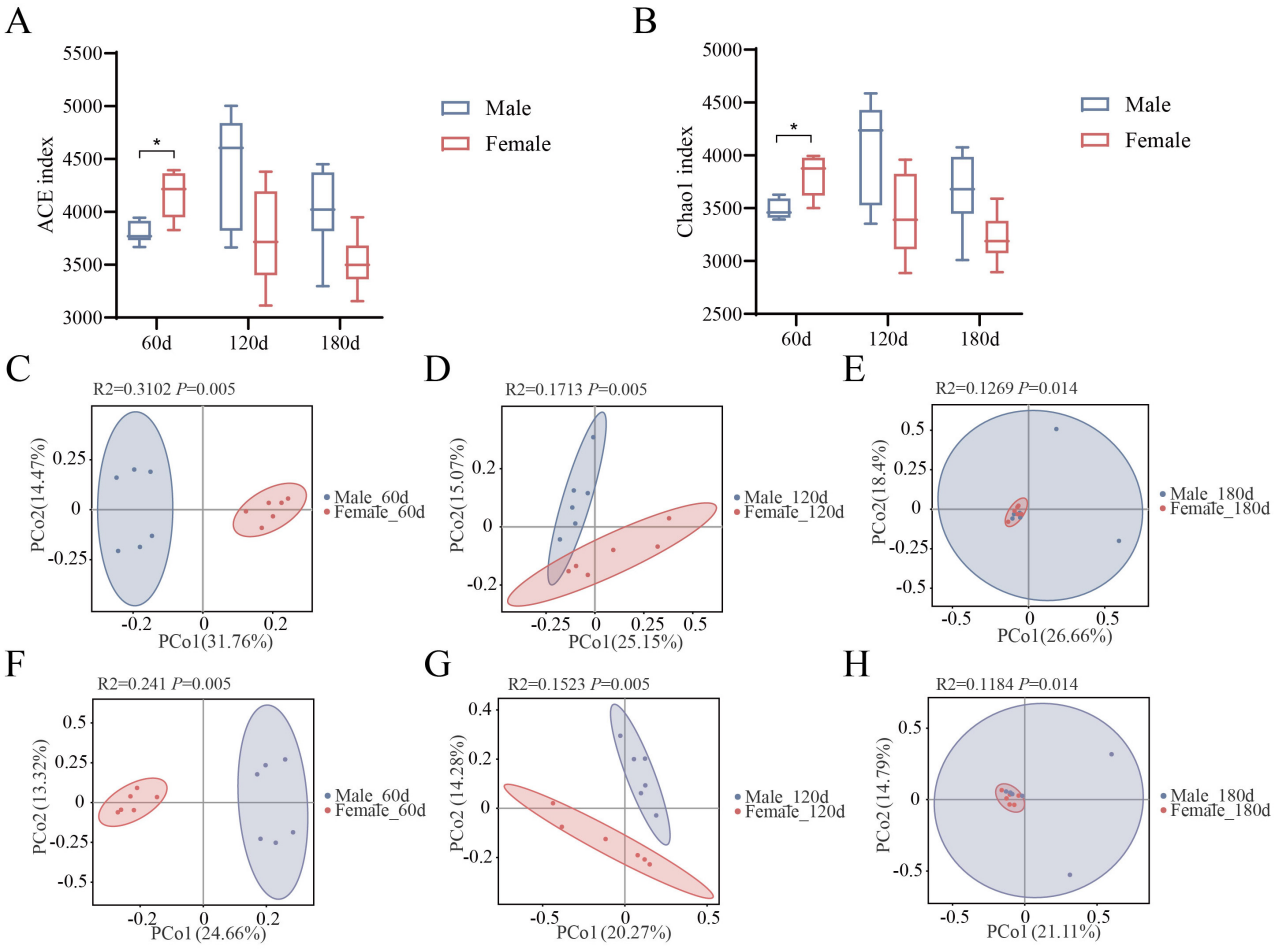
Comparison of gut microbial diversity between male and female Qiandongnan Xiaoxiang chickens. **(A)** ACE index and **(B)** Chao1 index; **(C-E)** PCoA based on Bray-Curtis distances at 60, 120 and 180 days of age; **(F-H)** PCoA based on Jaccard distance at 60, 120 and 180 days of age. *n* = 6 per sex per age group.

To investigate the effect of sex on gut microbial diversity, the alpha diversity of intestinal microorganisms in Qiandongnan Xiaoxiang chickens was evaluated using ACE and Chao1 indices. Two-way ANOVA revealed significant sex × age interactions for both indices (*P* < 0.05). Further post-hoc comparisons (Tukey's HSD) showed that at 60 days of age, both ACE and Chao1 indices were significantly higher in hens than in roosters (*P* < 0.05); whereas at 120 and 180 days, although both indices were lower in hens than in roosters, the differences did not reach statistical significance (*P* > 0.05) (Figures 2A, B). Beta diversity was assessed using principal coordinate analysis (PCoA) based on Bray-Curtis and Jaccard distances. Multi-factor PERMANOVA based on both distance matrices revealed significant effects of sex (Bray-Curtis: *P* = 0.024; Jaccard: *P* = 0.021), age (both *P* = 0.001), and their interaction (Bray-Curtis: *P* = 0.012; Jaccard: *P* = 0.014) on microbial community structure. PCoA plots showed clear separations between rooster and hen samples at each age (Figures 2C–H), and pairwise PERMANOVA with FDR correction further confirmed significant differences in bacterial community composition between sexes within each age (adjusted *P* < 0.05). These results indicate that sex significantly affects the gut microbial community structure of Qiandongnan Xiaoxiang chickens, and this effect exhibits dynamic changes with age.

### 3.5 Gut microbial community composition of Qiandongnan Xiaoxiangsickens

**Figure 3:**
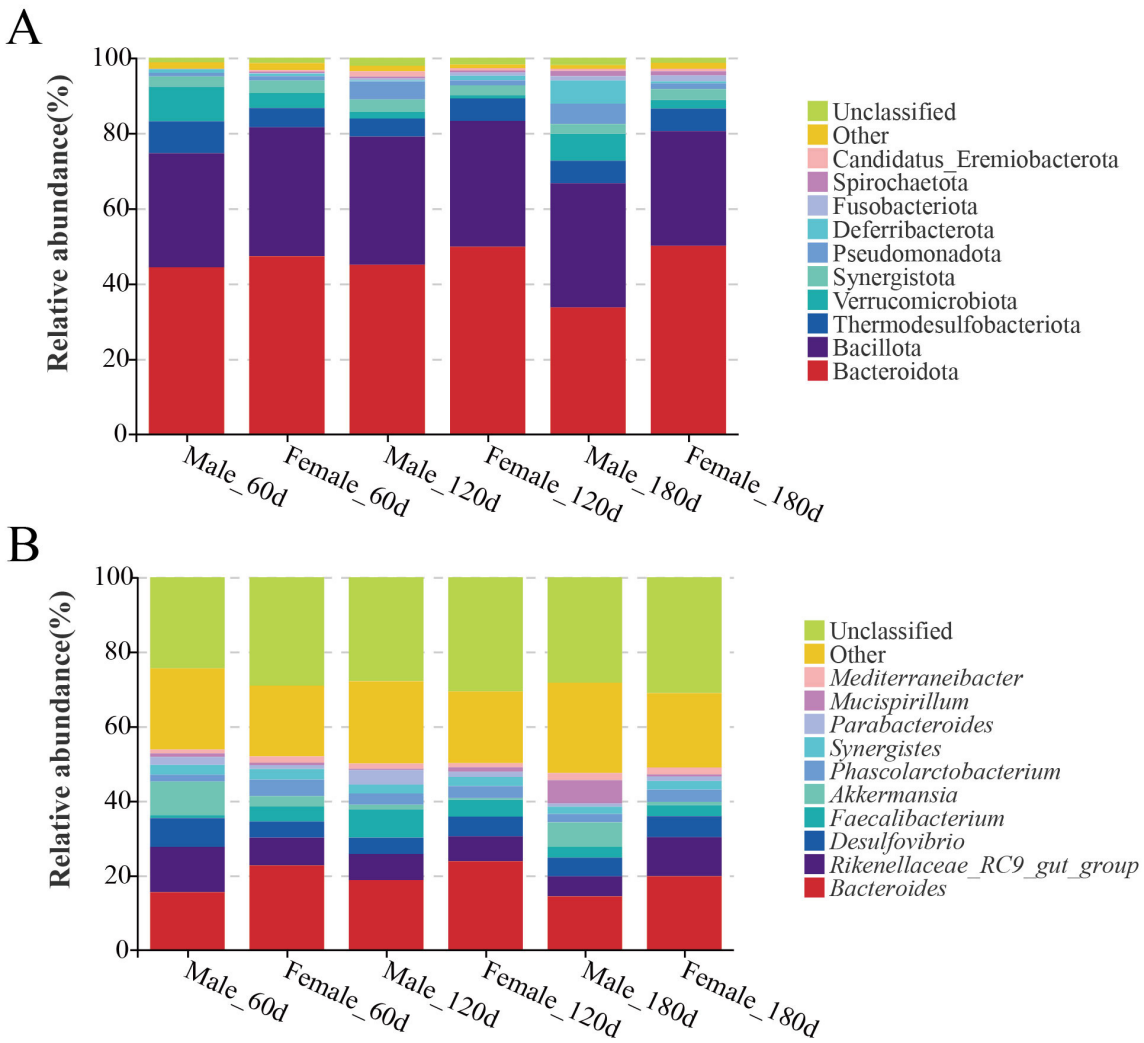
Relative abundance of gut microbial communities at the phylum **(A)** and genus **(B)** levels in male and female Qiandongnan Xiaoxiang chickens. *n* = 6 per sex per age group.

At the phylum level, the dominant phyla in both male and female Qiandongnan Xiaoxiang chickens across different ages were Bacteroidetes and Bacillota. The relative abundance of Bacteroidetes was higher in hens than in roosters at all three ages. For Bacillota, hens exhibited a higher relative abundance than roosters at 60 days of age, but lower abundances at 120 and 180 days of age. The relative abundance of Verrucomicrobiota in the intestines of hens was lower than that of roosters at all three ages (Figure 3A). At the genus level, *Bacteroides, Rikenellaceae_RC9_gut_group*, and *Desulfovibrio* were the dominant genera. The relative abundance of *Bacteroides* was higher in hens than in roosters at all three ages (Figure 3B).

### 3.6 Analysis of sex differences in gut microbiota of Qiandongnan Xiaoxiang chickens

**Figure 4:**
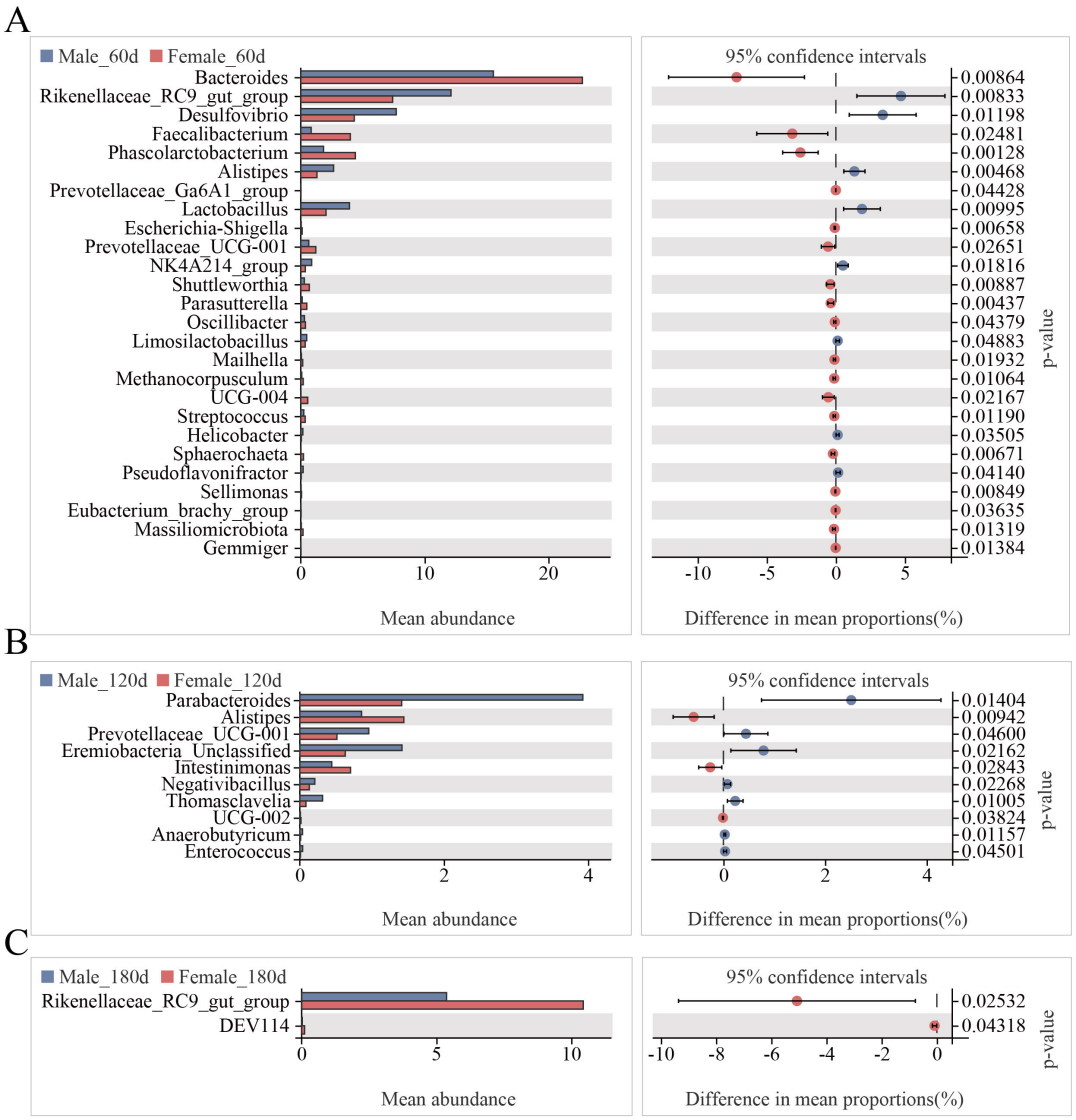
Comparison of differentially abundant gut genera in Qiandongnan Xiaoxiang chickens at different ages. **(A)** 60 days of age; **(B)** 120 days of age; **(C)** 180 days of age. *n* = 6 per sex per age group. Statistical analysis was performed using Welch's *t*-test.

At 60 days of age, the differences in gut microbiota between roosters and hens were most pronounced, with significant differences in the relative abundance of 26 genera. Roosters showed significant enrichment of 9 genera, including *Rikenellaceae_RC9_gut_group, Desulfovibrio*, and *Alistipes*, while hens exhibited significant enrichment of 17 genera, such as *Bacteroides, Faecalibacterium*, and *Phascolarctobacterium* (Figure 4A). By 120 days of age, the number of differentially abundant genera decreased to 10. Roosters displayed higher abundances of 7 genera, including *Parabacteroides* and *Prevotellaceae_UCG-001*, whereas *Alistipes, Intestinimonas*, and *UCG-002* were significantly enriched in hens (Figure 4B). At 180 days of age, 2 genera showed significant differences, with *Rikenellaceae_RC9_gut_group* and *DEV114* significantly enriched in hens. The sexual dimorphism of the gut microbiota in Qiandongnan Xiaoxiang chickens exhibited a dynamic evolution: the differences were most extensive at 60 days of age, gradually narrowed with increasing age, and by 180 days, only 2 genera remained significantly different (Figure 4C). This indicates that the shaping effect of sex on microbial community structure persists throughout the entire growth and development process, but its influence intensity varies with age.

### 3.7 Correlation analysis between genera and muscle phenotypes

**Figure 5:**
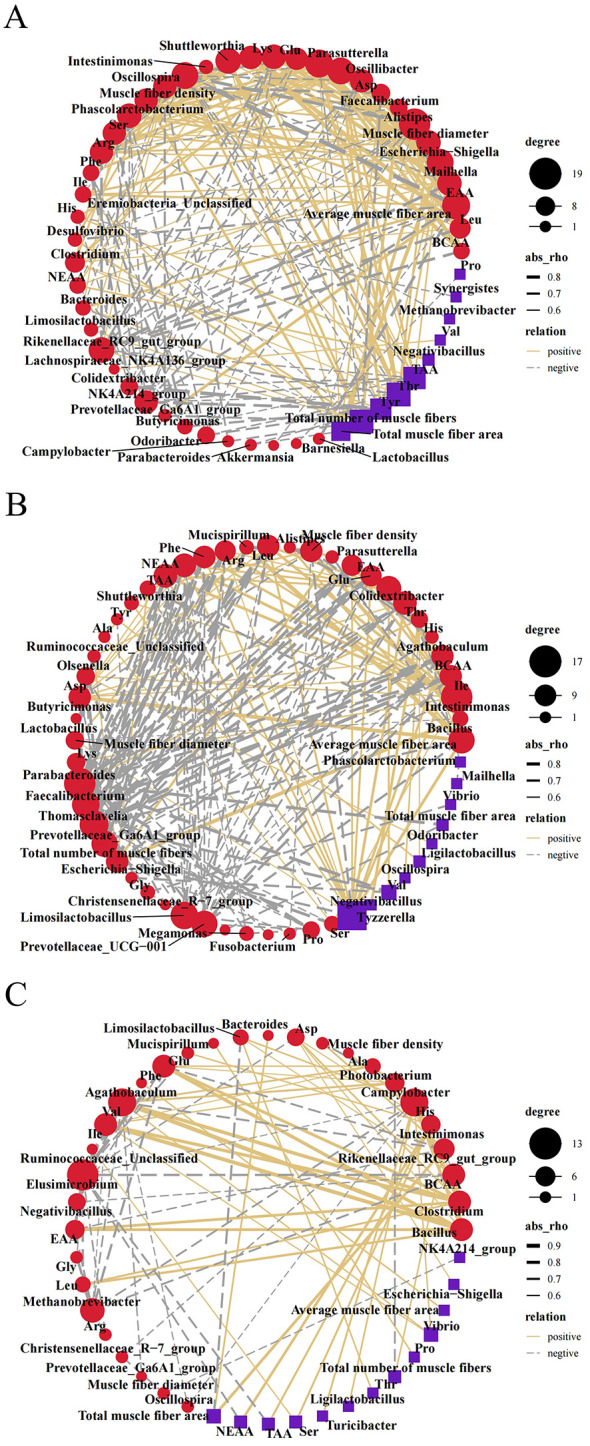
Correlation network diagram of differential genera and muscle phenotypes at 60 **(A)**, 120 **(B)**, and 180 **(C)** days of age.

At 60 days of age, extensive correlations were observed between gut microbiota and muscle traits. Genera such as *Prevotellaceae_UCG-001, Parasutterella*, and Oscillibacter showed significant positive correlations with multiple amino acids (e.g., BCAA, EAA, leucine, and phenylalanine) and muscle fiber diameter (rho>0.70, *P* < 0.05). In contrast, genera including *Alistipes, Rikenellaceae_RC9_gut_group*, and *Oscillospira* were negatively correlated with amino acid contents and average muscle fiber area (rho < −0.70, *P* < 0.05). Notably, *Phascolarctobacterium*, which was enriched in hens at 60 days, exhibited positive correlations with average muscle fiber area and total muscle fiber area (rho = 0.739 and 0.741, respectively, *P* < 0.05). At 120 days of age, the correlation pattern shifted. *Intestinimonas* and *Tyzzerella* were positively correlated with multiple amino acids (including BCAA, EAA, leucine, and isoleucine) and average muscle fiber area (rho > 0.70, *P* < 0.05), whereas *Faecalibacterium, Parabacteroides*, and *Thomasclavelia* showed negative correlations with various amino acids (rho < −0.70, *P* < 0.05). *Bacillus* was positively correlated with total muscle fiber number and fiber density (rho = 0.846, *P* < 0.05) but negatively correlated with average muscle fiber area (rho = −0.818, *P* < 0.05). At 180 days of age, the number of significant correlations decreased. *Bacillus* and *Clostridium* were positively correlated with amino acids including valine, isoleucine, BCAA, and phenylalanine (rho > 0.80, *P* < 0.05), while *Elusimicrobium* and *Methanobrevibacter* exhibited negative correlations with BCAA, EAA, valine, and leucine (rho < −0.70, *P* < 0.05). Additionally, *Rikenellaceae_RC9_gut_group* was positively correlated with total muscle fiber area and fiber density (rho = 0.769 and 0.664, respectively, *P* < 0.05), whereas *Campylobacter* showed negative correlations with fiber density and total muscle fiber number (rho = −0.676 and −0.660, respectively, *P* < 0.05) (Figure 5, Supplementary Table 2). Overall, the correlation patterns between gut microbiota and muscle traits exhibited dynamic changes with age, with the most extensive correlations observed at 60 days and gradually diminishing by 180 days, which is consistent with the trend of narrowing sex differences in both microbiota and muscle traits.

## 4 Discussion

This study systematically compared the differences in pectoral muscle fiber characteristics, amino acid content, and gut microbiota composition between male and female Qiandongnan Xiaoxiang chickens at 60, 120, and 180 days of age. It revealed sex-dependent dynamic changes in meat-quality-related traits and explored potential correlations between gut microbiota and these traits.

Muscle fibers are the basic functional units of skeletal muscle, and their number, size, and type composition directly determine meat tenderness, juiciness, and flavor characteristics (Zhang et al., 2024). This study found that at 60 days of age, the total number and density of muscle fibers were significantly higher in roosters than in hens, whereas the total area, average area, and diameter of muscle fibers were significantly greater in hens than in roosters. These results suggest that during early growth and development, roosters promote muscle growth mainly by increasing the number of muscle fibers, whereas hens tend to increase muscle weight by enlarging muscle fiber volume. This sexually dimorphic growth strategy may be closely related to differences in sex hormone levels. As age increased to 120 and 180 days, the sex differences in total muscle fiber number and density gradually disappeared, while only the total muscle fiber area remained significantly higher in hens than in roosters. These results indicate that the sex difference in muscle fiber number is prominent at early stage but diminishes with age, whereas the sex difference in muscle fiber area persists (James et al., 2025). Two-way ANOVA revealed no significant sex × age interaction for any of the muscle fiber traits (all *P* > 0.05), indicating that the sex effects were largely consistent across ages, and the observed attenuation of significant differences in individual indicators with age reflects a reduction in effect magnitude rather than a true change in sex-dependent regulation.

Amino acid composition is an important indicator for evaluating the nutritional value and flavor quality of meat (Liao et al., 2025). The results of this study showed that the sex difference in amino acid content of pectoral muscle in Qiandongnan Xiaoxiang chickens exhibited a dynamic pattern: at 60 and 120 days of age, hens were superior in most amino acids, whereas at 180 days, roosters became dominant. This reversal phenomenon may be related to differences in metabolic regulation between sexes at different growth stages (Suliman et al., 2023). During early growth and development, hens may be more inclined to allocate nutrients for protein deposition to meet rapid growth demands; after sexual maturity, nutrient allocation in hens may shift toward the reproductive system, resulting in relatively lower protein synthesis efficiency, while roosters continue to maintain a higher capacity for muscle protein deposition (Vignale et al., 2017; England et al., 2024). Two-way ANOVA confirmed significant sex × age interactions for most amino acids (*P* < 0.05), consistent with this dynamic reversal pattern. This study also found that at 120 days of age, histidine content was significantly lower in hens than in roosters, but this difference disappeared by 180 days, indicating that the sex difference in histidine is age-dependent and transient.

As important regulators of host metabolism, the composition and function of gut microbiota are closely related to muscle development (Yan et al., 2025). This study systematically analyzed, for the first time, the sex differences and dynamic changes of gut microbiota in Qiandongnan Xiaoxiang chickens at different ages. Alpha diversity analysis showed that at 60 days of age, the microbial diversity of hens was significantly higher than that of roosters, whereas at 120 and 180 days, the pattern reversed, with hens showing lower diversity than roosters. Beta diversity analysis further confirmed significant separation in microbial community structure between sexes at the same age (Lee et al., 2017). This dynamic change pattern exhibited a certain synchrony with the trend of sex differences in muscle fiber characteristics: at 60 days of age, both microbial diversity and muscle fiber indicators showed the most pronounced sex differences, which gradually narrowed with increasing age. In terms of microbial composition, hens exhibited a higher relative abundance of Bacteroidetes at all ages, whereas the abundance of Bacillota changed from being higher in hens than roosters at 60 days to lower in hens at 120 and 180 days. Previous studies have suggested that hormones may influence muscle traits via gut microbiota modulation, and that gut microbiota participates in sex-dependent glycan and lipid metabolism in broilers (Cui et al., 2021). These findings provide a broader context for our observational results, but direct mechanistic links remain to be established in Qiandongnan Xiaoxiang chickens.

This study found that the sexual dimorphism of gut microbiota exhibited a dynamic evolution: the differences were most extensive at 60 days of age (26 differentially abundant genera), gradually narrowed with increasing age, and by 180 days only 2 genera remained significantly different. This change may be associated with stage-specific physiological factors, including potential hormonal influences (Santos-Marcos et al., 2023). After sexual maturity, age-related changes in the intestinal environment may contribute to a new dynamic equilibrium of microbial structure (Koren et al., 2012). Similarly, in layer hens, the cecal microbiota maintained a constant Bacteroidetes-to-Bacillota ratio after 7 months of age, indicating a stable microbial equilibrium (Videnska et al., 2014). The decreasing number of differentially abundant genera with age paralleled the attenuation of significant sex differences in individual muscle fiber indicators, suggesting a potential temporal association between microbial community convergence and muscle trait maturation.

Correlation analysis revealed extensive associations between differential genera and muscle phenotypes. *Phascolarctobacterium*, enriched in hens at 60 days of age, was positively correlated with total muscle fiber area, while *Intestinimonas*, enriched in hens at 120 days, was positively correlated with multiple amino acids. These results suggest potential associations between these dominant genera and meat-quality-related traits in Qiandongnan Xiaoxiang hens during early and mid-growth stages, possibly involving amino acid accumulation and muscle fiber growth (Duan et al., 2024; He et al., 2025). Based on previous reports, *Phascolarctobacterium* and *Intestinimonas* are known as SCFA-producing genera, and their potential roles in modulating host metabolism have been investigated in other species (Bui et al., 2016; Ogata et al., 2019). However, it is important to note that these metabolic functions have been primarily characterized in humans and model organisms, and their relevance to chicken muscle physiology requires direct experimental validation. Therefore, the observed positive correlations should be interpreted as preliminary associations that warrant further investigation, rather than evidence of direct causation.

It is important to acknowledge several limitations of this study. First, the sample size (*n* = 6 per sex per age) is relatively small, which limits the statistical power for microbiome analysis and comparisons across multiple variables, and may increase the risk of false positive or false negative findings. Second, we measured muscle fiber characteristics and amino acid composition, which are important determinants of meat quality but do not constitute a comprehensive assessment. Classical meat quality traits such as pH, meat color, drip loss, shear force, and intramuscular fat were not evaluated. Therefore, conclusions regarding “meat quality” should be interpreted with caution, and the observed associations between microbiota and muscle phenotypes do not imply causation. Third, this study is limited to correlation analysis. Fourth, we did not measure sex hormones (e.g., estrogen, testosterone), microbial metabolites (e.g., short-chain fatty acids, bile acids), or conduct transcriptomic/functional analyses, which limits our ability to provide an in-depth mechanistic interpretation of the gut-muscle axis. Fifth, the basal diet differed between the starter and grower phases, which means that age-related changes may be partially confounded with dietary transitions. Although this is standard practice in poultry production, it should be considered when interpreting age effects. Future research incorporating larger sample sizes, direct meat quality measurements, metabolomics, hormone profiling, and functional experiments (e.g., fecal microbiota transplantation) is needed to further verify the causal role and specific mechanisms of gut microbiota in sex-dependent regulation of meat-quality-related traits. Sixth, 16S rRNA data were analyzed using 97% OTU clustering (USEARCH) rather than ASV-based methods. Thus, our results may be more conservative than ASV-based pipelines.

## 5 Conclusion

This study found that the pectoral muscle fiber characteristics, amino acid content, and gut microbiota composition of Qiandongnan Xiaoxiang chickens all exhibit significant sexual dimorphism, and this dimorphism dynamically evolves with age. Sex-associated differences in microbial community structure persist throughout the entire growth and development process, and correlations between microbial changes and muscle metabolic phenotypes were observed. This study provides a new microbiological perspective for understanding sex differences in meat-quality-related traits of local chicken breeds and offers a preliminary theoretical reference for sex-differentiated feeding management and genetic breeding of high-quality broilers. However, the correlational nature of this study and the absence of direct meat quality measurements (e.g., pH, color, drip loss) should be noted as limitations.

## Data Availability

The raw sequence data supporting the findings of this study are available in the NCBI database (https://www.ncbi.nlm.nih.gov), under BioProject accession number PRJNA1462278.
